# Sagittal Positional Changes of the Mandible Following Alignment and Levelling of Class II Division 2 Cases: An Observational Study in Decelerating Stages of Adolescent Growth Spurt

**DOI:** 10.7759/cureus.32653

**Published:** 2022-12-18

**Authors:** Shajin Shaik, Ganugapanta Vivek Reddy, Johnson Perala, Keerthipati Thejasri, Gowri Sankar Singaraju, Prasad Mandava

**Affiliations:** 1 Orthodontics and Dentofacial Orthopaedics, Narayana Dental College, Nellore, IND

**Keywords:** mandibular, malocclusion, sagittal, deep bite, condyle, class ii division 2

## Abstract

Introduction

The objective of this observational study is to compare the dental and skeletal changes that occur following the release of incisor locking in class II division 2 patients in the decelerating phase of the adolescent growth spurt.

Materials and methods

Lateral cephalograms of 17 subjects with skeletal class II and division 2 malocclusion, taken at the pre-treatment (T1) and post-leveling and alignment (T2) phases, were analyzed. All these patients were treated with non-extraction methods in the initial stage. A total of 25 skeletal and dental parameters, which included linear and angular measurements, were evaluated.

Statistical analysis

A paired t-test was used to compare the difference in the dimensional values between (T1) and (T2) points of the time period. The results were considered statistically significant at Bonferroni adjusted p<0.002.

Results

A statistically significant positional change was noted in the condylar position both in the vertical and sagittal directions, resulting in sagittal changes of the mandible in the forward direction. The deep bite was relieved by vertical changes in the dental structures in both the posterior and anterior segments. Growth changes in the vertical direction were also noted but not conclusive.

Conclusions

There was a definite horizontal shift of the mandible, improving maxillo-mandibular relations following the unlocking of the bite in class II division 2 patients. This shift is mostly attributed to the condylar repositioning in the forward and downward directions.

## Introduction

Class II division 2 (div 2) malocclusion is a unique subclass of class II malocclusion, characterized by distinctive dental and craniofacial features that differentiate it from other malocclusions. Angle's class II div 2 malocclusion is also called "cover-bite" or "Deckbiss" in its early German descriptions because of its extremely deep overbite with skeletonized facial hypo divergence, maxillary incisor retroclination, mandibular dentoalveolar retrusion, excessive bony chin projection, and 100% overbite, covering at least one mandibular incisor in occlusion [1]. Due to the retroclination of maxillary incisors, overbite is increased, which is the most damaging feature of this malocclusion and makes it overly complex when superimposed on class II skeletal base [2]. According to McNamara, the most important feature of class II malocclusion is mandibular retrusion rather than maxillary prognathism [3], and hence measures should be taken to express the full growth of the mandible depending upon the age.

The clinical management of class II division 2 malocclusion still remains a daunting task that poses diagnostic, treatment, and retention challenges for the orthodontist. It has been suggested that the natural forward movement of the mandible (between points B and Pog) can be inhibited by either a deep incisal overbite or a significant forward rotating pattern of mandibular growth, and in many subjects, both are expected together [4]. Given the limited evidence available in the orthodontic literature, it is generally accepted that unlocking the mandible in class II div 2 deep bite malocclusions allows mandibular growth to be expressed in a more anterior direction, thereby allowing correction of the disto-occlusion [4,5,6,7]. 

The mandibular position changes occur after the leveling and alignment phase after overjet correction in class II div 2 malocclusions, especially with patients in the active growth phase [4]. Ricketts believed that it was important to "unlock" the deep bite by advancing the upper incisors, which would then resemble a class II division 1 malocclusion that could be treated with more dental changes instead of skeletal changes [8]. Based on these postulates, this study was designed to explore whether a change in the inclination of retroclined maxillary incisors by the fixed appliance in class II div 2 patients with decelerating growth phases can bring about a change in the spatial and positional mandibular skeletal structures.

## Materials and methods

A cross-sectional observational study based on retrospective data measured at two different time points. The sample for the study was drawn from pre- and post-alignment treatment records of class II div 2 cases registered and treated as outpatients in the Department of Orthodontics at Narayana Dental College, Nellore, India, between the years 2012 and 2017. Ethical approval for the study was obtained from the Institutional Ethics Committee of Narayana Dental College and Hospital, Nellore (NDC/IECC/ORTHO/DISS/12-18/03). Data collected included a patient registration card with demographic details, a patient outpatient card and case sheet, patient study models, hand-wrist x-rays, lateral cephalograms, and an orthopantomogram (OPG).

A manual search of the permanent patient database identified all subjects seen for initial records between August 1, 2012, and July 31, 2017. A total of 28 records of class II division 2 patients were selected based on inclusion and exclusion criteria. Mandibular crowding of no greater than 5 mm with a full complement of teeth was taken up for the study. Based on a previous study on the local population [9], patients in the age group of 13-16 years with Cervical Vertebral Maturation Index (CVMI) stages 4 and 5 [10] and Skeletal Maturation Index (SMI) stages 6, 7, and 8 of hand-wrist radiographs with an MP3 cap to Dp3-U [11], which characterize the decelerating growth phase, were included. Patients with severe transverse problems, pain, and clicking sounds in the temporomandibular joint were excluded from the study. The lateral pretreatment cephalograms were studied, and class II skeletal bases were assessed by Wits appraisal [12]. An overbite of more than 50% with maxillary incisor retroclination and an overjet of less than 2 mm were considered. Horizontal mandibular growth tendency, as demonstrated by an MP-SN plane angle of 32° or less, was included. All the radiographs were taken with the Cephalostat (model no. MR05, type 84086511; Villa Sistemimedicali, Italy). The magnification error was found to be 8%, which is clinically acceptable. The lateral cephalograms of 28 selected patients taken at T1 (before initiation of treatment) and T2 (after alignment, leveling, and settling) were evaluated.

Up until the stage of alignment, all cases were treated by the fixed appliance using non-extraction methods. The treatment protocol for the selected subjects consisted of a fixed orthodontic treatment with Standard MBT (Masters series; American Orthodontics) brackets with a 0.022 × 0.028 slot, bonded on the upper and lower arches to align the teeth. Alignment and incisor inclination correction were brought about by using 0.014-in. NiTi archwire and 0.018-in. NiTi, followed by 17- × 25-in. NiTi and 19- × 25-in. NiTi arch wires, respectively. The upper and lower arches were stabilized using the 19 × 25 SS wire. The transpalatal arch and lingual arch were used in the upper and lower arches, respectively. T2 records were taken after initial alignment based on the following criteria: 50% of the initial overbite correction, correction of the smile curve with only 0-2 mm remaining, and well-aligned arches to confirm the completion of the alignment and leveling phase. This was followed by a settling phase of two months, during which no further changes in overbite and overjet were observed clinically. To reach this stage of settlement, the average treatment time was six months.

The cephalograms were analyzed for skeletal and dentoalveolar changes from T1 to T2 using various cephalometric parameters (Table 1 and Table 2). The landmarks, line angles, reference lines, and angular measurements were adapted from previous studies after slight modifications [2,13]. Absolute distances were measured from landmark to landmark. All the measurements were measured as coordinates, with the respective points projected onto the SNver or SNHor lines.

**Table 1:** Landmarks and reference planes used in the study N: nasion; S: sella; Ba: basion; Ptm: pterygomaxillare; Ar: articulare; A: point A; B: point B; ANS: anterior nasal spine; PNS: posterior nasal spine; Cd: condylion; Gn: gnathion; Go: gonion; Pg: pogonion; U6: upper first molar; L6: upper first molar; ILS: incisor superius; Ili: incisor inferius

Landmarks	
Nasion (N)	Anterior limit of the nasofrontal suture.
Sella (S)	Center of sella turcica
Basion (Ba)	The lowest and posteriormost point on the anterior contour of the foramen magnum.
Pterygomaxillare (Ptm)	The point of intersection of the hard palate, soft palate, and pterygopalatine fissure.
Articulare (Ar)	The intersection point of the posterior border of the condyle and the posterior cranial base.
Point A (A)	The deepest point on the midline between the anterior nasal spine and the alveolar crest is between the two central incisors.
Point B (B)	It is the deepest point between the alveolar crest of the mandible and the mental process.
Anterior nasal spine (ANS)	The tip of the anterior median sharp bony process of the palatine bone.
Posterior nasal Spine (PNS)	The tip of the posterior nasal spine of the palatine bone.
Condylion (Cd)	A point on the contour of the condyle is obtained by bisecting the angle formed by tangents to the upper and posterior borders of the condyle, the tangents being parallel to the sagittal and vertical axes of the face, respectively.
Gnathion (Gn)	The deepest and anteriormost point of the symphysis. This is obtained by bisecting the angle between the mandibular plane and the tangent to the anterior border of the mandible.
Gonion (Go)	The point on the posterior contour of the mandible is obtained by bisecting the angle between the mandibular plane and the tangent to the posterior border of the mandible.
Pogonion (Pg)	The most prominent point of the symphysis is measured in relation to the mandibular plane (MP).
U_6_	The midpoint of the occlusal surface of the maxillary first permanent molar.
L_6_	The mid-point of the occlusal surface of the mandibular first permanent molar.
Incisor superius(IL_S)_:	The inferiormost tip of the maxillary incisor
incisor inferius(Il_i)_:	The superiormost tip of the mandibular incisor
II. reference planes	
SN:	The line between the sella and the nasion.
SNP:	A perpendicular line is drawn to the sella and a nasionplane dropped from the sella.
SN_hor_	A horizontal line is drawn at an angle of 8 degrees to the SN line
SN_ver_	A vertical line is drawn perpendicular to the SN_hor_ line
NP:	Nasal plane: The line between the posterior nasal spine (PNS) and the anterior nasal spine (ANS).
MP:	Mandibular plane: The tangent to the lower border of the mandible through the gonion and the gnathion
IL_S_:	The line from the incisor superius through the apex of the upper central incisor.
Il_i_:	The line from the incisor inferius through the apex of the lower central incisor.

**Table 2:** Parameters and method of measurement N: nasion; S: sella; Ba: basion; Ptm: pterygomaxillare; Ar: articulare; A: point A; B: point B; ANS: anterior nasal spine; PNS: posterior nasal spine; Cd: condylion; Gn: gnathion; Go: gonion; Pg: pogonion; U6: upper first molar; L6: upper first molar; ILS: incisor superius; Ili: incisor inferius

Skeletal-linear measurements	
B-A:	The difference in the horizontal distances between points B-SNP and A-SNP. Measures the sagittal-basal jaw relationship.
Pg-A:	The difference in the horizontal distances from points Pg and A to the SN perpendicular. measures the sagittal jaw relationship of the chin to the maxilla.
Go-ptm:	The sagittal distance between the posterior borders of the mandibular and maxillary corpora to SNP.
B-pg:	Sagittal distance between point B and the pogonion. The difference in the horizontal distances from points B and Pg to the SN perpendicular. Measures the inclination of symphysis and thickness of the chin.
Cd-S:	Sagittal distance between the condylion and the sella. The difference in the horizontal distances from points cd and S to SN perpendicular. Measures the sagittal position of the condyles.
S-cd:	The vertical distance between the sella and the condylion projected on the SN_ver_ line. Measures the vertical position of condyles.
Ptm-A:	The separation between the pterygomaxillary fissure and the A-point. Measured along the nasal plane (NP). Measures the length of the maxillary corpus.
Go-B:	The distance between the gonion and the B-point. Measures the length of the mandibular corpus minus the mental process.
Ar- Go	The vertical distance between the articulare and the gonion. Measures the ramal length projected on SN_ver_
Skeletal-angular measurements	
SNA:	An angle formed between the SN plane and a line drawn through the nasion to point A. Measures the maxillary prognathism.
SNB:	An angle formed between the SN plane and a line drawn through the nasion to point B. Measures the mandibular prognathism.
SN-pg:	An angle formed between the SN plane and a line drawn through the nasion to the pogonion. Measures the position of the chin in relation to the anterior cranial base.
SN-Go:	An angle formed between the SN plane and a line drawn through the nasion to the gonion. Measures the sagittal position of the gonion in relation to the anterior cranial base.
MP-SN:	The angle between the mandibular plane and SN plane. Measures the growth rotation of the mandible.
Facial axis (FA) angle:	The angle formed between the basion-nasion and the Ptm- gnathion. Measures the position of the chin.
Dental-angular measurements	
U_1-_SN	The angle between the long axis of the upper central incisor and SN plane. Measures position of the maxillary incisors.
L_1-_MP:	The angle between the long axis of the lower central incisor and the MP plane. Measures the position of the mandibular incisors.
IL_S _- IL_i_:	The angle between the upper central incisor and the lower central incisor (interincisal angle).
IL_S- _NP	Distance between the upper incisor edge to the nasal floor (NP) projected on SN_ver. _Measures the upper anterior dentoalveolar height
IL_i-_MP	Distance between the lower incisor edge to the mandibular plane (MP) projected on SN_ver. _Measures the lower anterior dentoalveolar height.
U_6_-NP	Distance between point U6 and nasal floor (NP). Measured along the perpendicular to the nasal floor projected on SN_ver_. Measures the upper posterior dentoalveolar height
L_6_-MP:	Distance between point L6 and mandibular plane (MP). Measured along the perpendicular to the mandibular plane projected on SN_ver_. Measures the lower posterior dentoalveolar height
Overjet	The horizontal distance between IL_S _and IL_i _projected on SN_hor._
Overbite	The vertical distance between IL_S _and IL_i _projected on SN_ver._
Molar relation	The horizontal distance between U6 and L6 projected on SN_hor._

**Figure 1:**
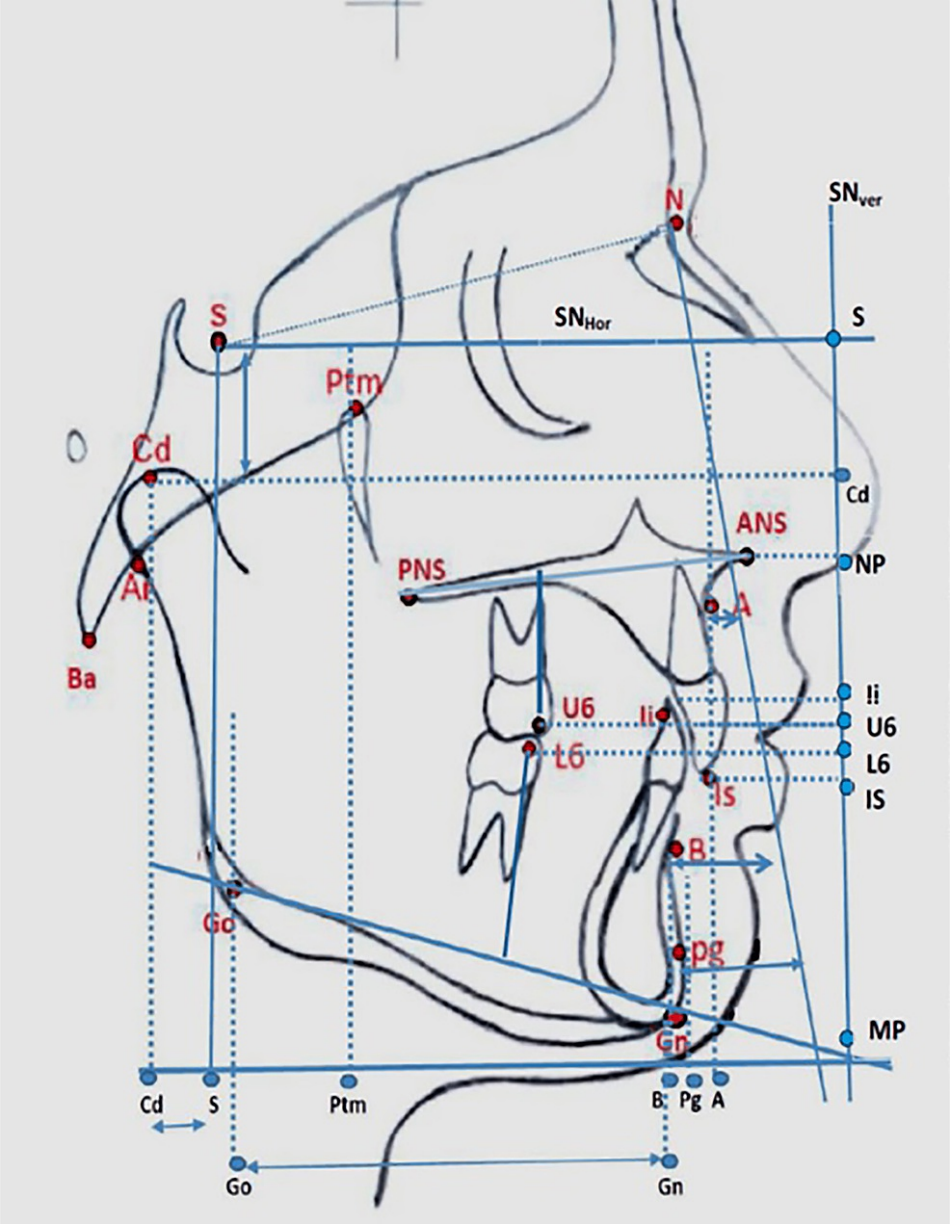
The landmarks and reference planes used in the study. The linear parameters measured in the study (refer to Table 1 and Table 2 for descriptions) N: nasion; S: sella; Ba: basion; Ptm: pterygomaxillare; Ar: articulare; A: point A; B: point B; ANS: anterior nasal spine; PNS: posterior nasal spine; Cd: condylion; Gn: gnathion; Go: gonion; Pg: pogonion; U6: upper first molar; L6: upper first molar; ILS: incisor superius; Ili: incisor inferius

**Figure 2:**
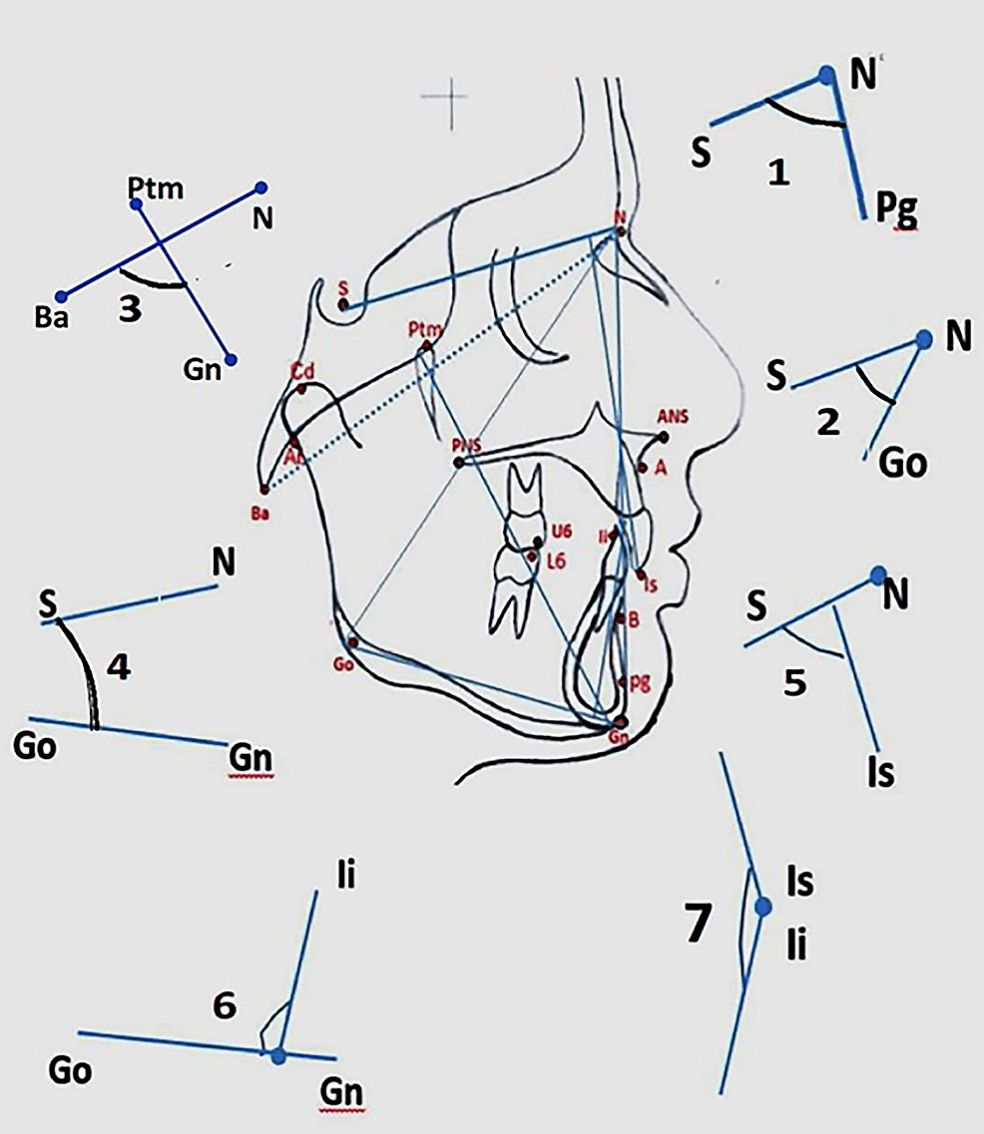
Angular parameters measured in the study. (1) S-N-Pg; (2) S-N-Go; (3) Facial axis angle; (4) SN-Is; (5) SN-MP; (6) Li-MP; (7) Is-Il (refer to Table 1 and Table 2 for descriptions). N: nasion; S: sella; Ba: basion; Ptm: pterygomaxillare; Ar: articulare; A: point A; B: point B; ANS: anterior nasal spine; PNS: posterior nasal spine; Cd: condylion; Gn: gnathion; Go: gonion; Pg: pogonion; U6: upper first molar; L6: upper first molar; ILS: incisor superius; Ili: incisor inferius

The sagittal measurements were done on the SNHor line with lines dropped parallel to the vertical plane, and all the vertical measurements were done with lines dropped parallel to the horizontal plane from a given point. (Figure 1, Figure 2).

All the serial lateral head radiographs were hand-traced, and in the final run, only 17 records were analyzed. The 11 records were excluded as these patients were treated with a fixed anterior bite plane. All the measurements were done by the primary investigator (SS) and calibrated with those of an experienced person (GVR). To evaluate accuracy and reproducibility, six pretreatment and post-treatment cephalograms were randomly selected and remeasured four weeks after the initial measurements. The reproducibility of the recorded measurements was evaluated by the Dahlberg formula test, which showed a reproducibility of 99% for linear measurements with an error of 0.02 mm and 98% reproducibility for angular measurements. All measurements were done with a digital caliper (a TOLEXO electronic digital vernier caliper with an LCD display screen) with an accuracy of 0.01 millimeters and a protractor with 0.5-degree gradings. All the readings were rounded off to the next nearest mm or degree.

All the linear measurements in millimeters and all the angular measurements in degrees were entered as quantitative continuous data on a Microsoft Excel 2010 sheet and then statistically analyzed with the software Statistical Package for the Social Sciences (IBM SPSS version 25 for Windows, IBM Corp., 2017 Armonk, NY). The normality of the data was assessed by the Shapiro-Wilk test, and basic descriptions were presented in the form of the mean and standard deviation. The Student's paired t-test for each of the parameters was used to detect treatment-related changes between the T1 and T2 stages. The Bonferroni correction was calculated to adjust the p-value for each of the 25 parameters tested on one set of data. An adjusted significance level of 0.002 was calculated to neutralize the risk of the increased possibility of type-I error.

## Results

A final analysis of 17 records with 10 males and seven females was included. The mean age of the males was 15.63 + 1.3, and the mean age of the females was 14.11 + 1.54. However, the data was not analyzed gender-wise.

A total of 25 parameters were analyzed in the study (Table 3). The Shapiro-Wilk test for normality of the data for pre-treatment and post-treatment cephalometric values showed that the values are centered over the mean, and a paired t-test was used for the analysis of the data between the pre-treatment and post-treatment. The difference for all the parameters was found to be statistically significant (p= 0.0001) except for B-Pg (p= 1.00) and FA angle (p= 0.8).

**Table 3:** Comparison of pre-treatment and post-treatment values of parameters measured: paired Student's t-test All p-values were adjusted after the Bonferroni correction. **p<0.002- significant; ^ns^p>0.002-non significant. N: nasion; S: sella; Ba: basion; Ptm: pterygomaxillare; Ar: articulare; A: point A; B: point B; ANS: anterior nasal spine; PNS: posterior nasal spine; Cd: condylion; Gn: gnathion; Go: gonion; Pg: pogonion; U6: upper first molar; L6: upper first molar; ILS: incisor superius; Ili: incisor inferius

Parameter	Pre-treatment	Post-treatment	Difference (post-pre) (+) or (-)	t-value	p-value
	Mean+SD	Mean+SD			
Skeletal-linear measurements (mm)					
B-A	8.41+2.79	6.88+3.3	-1.53	5.60	0.0001**
Pg-A	7.47+3.59	6.11+3.69	-1.354	5.01	0.0001**
Go-Ptm	25.05+3.20	23.52+3.69	-1.53	5.60	0.0001**
B-Pg	2.29+1.74	2.64+1.32	0.37	0.00	1.000^ns^
Cd-S	12.75+2.5411	.09+2.62	-1.66	6.81	0.0001**
S-Cd	13.23+4.09	14.91+3.95	1.68	6.81	0.0001**
Ptm-A	37.11+4.87	38.52+4.98	1.41	4.95	0.0001**
Go-B	54.64+4.71	56.11+4.99	1.47	4.92	0.0002**
Ar-Go	40.88+3.31	41.58+3.42	0.71	4.24	0.0006**
Skeletal-angular measurements (degrees)					
SNA	80.82+1.54	81.82+1.79	1.0	4.31	0.0005**
SNB	76+2.02	78.82+1.72	2.82	7.41	0.0001**
SN-Pg	80.64+3.44	81.88+3.35	1.24	3.77	0.0016**
SN-Go	41.64+3.0	40.05+3.18	-1.59	6.15	0.0001**
MP-SN	23.35+3.89	25.05+3.99	1.7	6.36	0.0001**
FA angle	89.88+3.10	90.17+1.38	0.29	0.21	0.8291^ns^
Dental-angular measurements (degrees)					
U1-SN	97.59+4.03	114.70+5.7	17.1	11.32	0.0001**
L1-MP	86.35+ 2.34	93.71+ 2.49	7.35	14.10	0.0001**
Ils-ili	151.11+10.5	132.52+5.28	-18.59	11.13	0.0001**
Dental-linear measurements (mm)					
IL_S_- NF	26.41+3.01	22.94+2.77	-3.47	9.52	<0.0001**
IL_i_- MP	39.41+2.93	37.67+ 3.08	1.76	9.66	<0.0001**
U6-NF	22.52+ 3.11	23.11+ 3.27	0.59	4.77	<0.0001**
L6- MP	22.47+3.20	24.17+3.24	1.74	7.13	<0.0001**
Overjet	1.5+0.6	5.2 + 0.84	3.26	12.61	<0.0001**
Overbite	6.35+1.27	3.82+1.13	2.53	11.06	<0.0001**
Molar relation	3.29+1.92	0.76 + 1.88	-2.53	14.54	<0.0001**

## Discussion

Earlier studies indicated that the unlocking of the distally held mandible by the retroclined maxillary central incisors in class II Div 2 cases through routine treatment procedures enhances the forward growth of the mandible [4,5,6], with a mean difference of approximately 1.5 mm/yr between treated subjects which was significantly greater than the untreated controls. Taylor [5] and Erickson [6] recommended early treatment of this malocclusion. Most of these studies included a wide range of age groups for analysis. To date, no investigation has identified or quantified the growth changes resulting from unlocking the bite in age groups with minimum growth potential remaining. Therefore, the purpose of this study was to assess the sagittal changes or repositioning of the mandible associated with unlocking the incisor inclination and deep bite in patients with class II div 2 who were treated with comprehensive fixed orthodontic treatment and were in the decelerating phase of the pubertal growth spurt. The average period of evaluation was 6 months between T1 and T2 radiographs.

The linear and angular parameters that depict the maxilla showed a slight forward growth of the maxilla. There was a slight increase in SNA angle (1.00) during this period. This is in tune with the findings of earlier studies [7,14] which found an increase of 1.84º and 1.19º of SNA after alignment in class II div 2 patients. It is most likely due to the remodeling of point A that occurs during the correction of the inclination of the upper incisors. At the same time, the linear increase of the maxillary corpus dimension is found to be 1.41 mm as measured by the Ptm-A distance.

During this phase of treatment, there was a mandibular base shift with SNB in the mesial direction, with a difference in the mean value of 2.82 degrees between pre-treatment and post-treatment. This pronounced mandibular shift may aid in correcting the class II skeletal pattern as there is a decrease in angle ANB of 1.8º in the present study. This finding correlates with the study of Binda [15], which showed a mean increase in SNB of 0.5º and was also confirmed in the recent study by Raghav [7] where there was a resultant decrease in ANB (1.36º, p<0.01*) between pre- and post-alignment and leveling phases. However, their study was not age-specific with regard to the class II division 2 patients. There was an improvement towards a favorable maxillo-mandibular relationship, as indicated by the angle Δ ANB of 1.8 degrees and the linear parameters Δ B-A (-1.532, p-0.0001), ΔPog-A (-1.354, p-0.0001), and ΔGo-Ptm (-1.53, p-0.0001).

The amount of mandibular growth was measured by the total distance from the articulare to the pogonion, represented by Ar-Go, Go-B, and B-Pg. The corpus length as measured by Go-B (Δ1.47mm, p-0.0002) and Ar-Go (Δ 0.71mm, p-0.0006) was found to be statistically significant. However, the changes in the chin region were not found to be significant statistically as represented by B-Pg (Δ 0.31mm, p-1.00). The Ar-Go indicates the relocation of the ramus or the condylar changes. The findings indicate that the prominence of the chin did not increase during this period. Thus, one might infer that the increase in Ar to Pg during treatment is the result of unlocking the mandible, or it may be only a temporary shift.

The growth direction represented by the angle SN-Go demonstrated a decrease of the gonial angle Go (Δ -1.59degrees, p-0.0001) during the aligning and leveling phase. Despite this sagittal advancement of the gonion, the angle MP-SN exhibited a mean increase of 1.7 degrees, indicating a vertical growth pattern. This finding is in line with the study of Ghobashy [16], where the children with class II div 2 malocclusion in the age group of 15-18 years treated with fixed orthodontic appliances had an increase of 2.5 degrees in the vertical mandibular axis opening in a clockwise direction. The findings of the present study are also in line with the study of Raghav [7] done in the age range of 17-24 years with fixed orthodontic therapy, where there is an increase in the SN-pog, which serves as a prognostic parameter for forward mandibular repositioning. In our study, the SN-Pg increased by a mean value of 1.24 mm.

During the same period, there was also a clinically significant increase in the height of the ramus as represented by the Ar-Go distance (Δ0.71 mm, p=0.0006), indicating vertical condylar growth. The relative anteroposterior position of the chin in relation to the cranial base as assessed by the FA angle also increased by a statistically non-significant mean (Δ0.29 degrees, p-0.829). This implies that the basic pattern of horizontal growth is not changed, and the increase in MP-SN angle may be attributed to the bite-opening mechanisms that resulted in the extrusion of the posterior teeth. It was reported in the literature that the smaller the gonial angle and the more vertical the condylar growth direction, the greater the likelihood that such a rotation of the mandible occurs. It follows that the bite-opening therapy favored vertical facial development, which was negated by the anterior rotational growth of the mandible. This is to be considered carefully during the treatment plan, as it was shown by Binda [15] that post-retention of class II div 2 cases has a high correlation between ramus growth and the decrease in the mandibular plane angle that had occurred during the correction of the deep bite.

There were significant changes in all the dental measurements. The mean bite opening during this phase of treatment was found to be 2.53 mm, with an increase in overjet of 3.26 mm. The dental parameters assessed in the anterior region exhibited an increase in the angle U1-SN by a mean value of 17.1 degrees (p-.0.0001), indicating maxillary incisor proclination, and the L1-MP revealed that the lower incisors are also proclinated by an average of 7.35 degrees to the mandibular plane (p-.0.0001). There was also a decrease in the interincisal angle, Ils-ili, by a mean value of 18 degrees. The reduction in deep bite during leveling was due to the extrusion of the upper posterior teeth (0.59 mm) and, in particular, the lower posterior teeth (1.74 mm), and the difference was statistically significant (p<0.0001). The relative perpendicular distance from the upper incisors to the palatal plane (ILS-NF) is decreased by -3.47 mm, and at the same time, the lower incisors are relatively intruded by 1.74 mm, as indicated by ILi-MP. There was a sagittal change in the molar relationship, and the lower molars moved mesially towards a class I relationship by 2.53 mm. The dental changes observed in the study are concordant with the treatment changes that occur during the treatment of class II div 2 cases from the studies available in the literature [7,16,17].

There was a statistically significant increase in the horizontal anterior and vertical repositioning of the condyle in the glenoid fossa. A mean horizontal shift of the condyle ( Cd-s) by -1.62 mm in the forward direction and an equal increase in the vertically downward direction (S-Cd) of 1.68 mm were observed and were statistically significant (p=0.0001). This correlates with the previous study [7] which concluded that there is a downward and forward shift of condyle observed in 36% of the subjects during the unlocking phase in class II div 2 subjects. The shift in the condyle position indicates that these subjects might have forced retrusion and a backward path of closure.

The results of the present study have certain clinical implications. The suggestion of Cleall and Begole [18] from their study on class II div 2 patients stated that the molar extrusion should be kept minimal except for that required to offset the horizontal growth tendency, which was also reflected in our findings. Buccal segments extruded beyond a certain limit are prone to relapse as the continued growth of the ramus favors anterior growth rotation, and any mismatch between the molar extrusion and ramus growth may lead to relapse of the deep bite [15].

The type of facial growth and age of the patient should be kept in mind when unlocking the mandible [4]. In the present study, there was a definite change in point B towards the improvisation of the maxilla mandibular relations. Both the cumulative linear (Ar-Go and Go-B) and angular values (SNB) as related to point B were increased by 2.5 mm and 2.8 degrees, respectively, during this study period in patients with a decelerating pubertal growth stage by unlocking the deep bite in div 2 cases by advancing the upper incisors, which would then convert to a Class II div 1 malocclusion that could be treated with more dental alterations instead of skeletal alterations. This includes either distalizing the dental arch, advancing the lower arch, or considering both. The extraction of only the upper four premolars should be postponed in these patients until the point where no more shift in point B is seen after unlocking of the mandible, particularly in mesofacial patterns [4], who may exhibit greater forward movement of the mandibular dentoalveolar with normal mandibular growth after incisal bite opening as less maxillary retraction is necessary for this patient group.

Another thoughtful evaluation to be done is regarding the position of the roots of the upper incisors after alignment and leveling so as to place them for good incisal guidance [19]. The slope of the palatal plate should be evaluated as the change in inclination may end up contacting the palatal cortical plate and hence the consequential root resorption.

Another interesting finding of the study was the linear increase in the maxillary apical base size by 1.41 mm despite a PG-A linear measurement showing a decreased value of -1.35. This should be kept in mind in formulating a treatment plan, as the maxillary apical base size was the strongest predictor of occlusal correction in class II div 2 [20]. The implicit implication of this finding is that some form of holding back the maxilla should be included in the treatment plan.

A significant change in lip pressure, especially lower lip pressure, was found to increase after protrusion and decrease after retention [21]. This also has to be kept in mind while proclining the upper and lower teeth during the correction of class II div 2 patients.

Limitations

The findings of this study should, however, be applied clinically with caution. The study is limited to patients in the decelerating stage of the adolescent growth spurt. The findings were limited to the post-aligning phase. The difference between male and female patients was not delineated for the amount of mandibular growth. The changes brought about due to growth and treatment mechanics were not differentiated. Multicollinearity exists due to the high correlation between two or more explanatory variables in the study, and thus a multiple regression cannot be constructed in this study.

## Conclusions

The release of vertical and sagittal incisor locking during the alignment and leveling phase in class II div 2 patients with a decelerating growth phase results in a forward displacement of the apical base of the mandible, mainly due to simultaneous position changes of the condyle. Nevertheless, a class II relationship can be corrected if an ideal maxillary arch form exists. The condylar repositioning and dental changes contributed to the leveling of the curve of Spee during this phase of treatment.
